# Osteogenesis imperfecta: an unusual presentation

**DOI:** 10.11604/pamj.2023.46.14.40948

**Published:** 2023-09-11

**Authors:** Hardik Patel, Sandeep Shrivastava

**Affiliations:** 1 Department of Orthopaedics, Datta Meghe Institute of Medical Sciences (DU), Sawangi, Wardha, Maharashtra, India

**Keywords:** Osteogenesis imperfecta, brittle bone disease, glass bone disease

## IMAGE IN MEDICINE

Osteogenesis imperfecta (OI), also known as brittle bone disease or “glass bone disease,” is a genetic disorder that affects the production of collagen and causes bones to be fragile and easily fractured. It is very rare condition, with varying degrees of severity, and present from birth. Its estimated incidence is around 1 in 100,000 live births. Here, we report a rare presentation of osteogenesis imperfecta type IV. A 7-year-old male child was brought to the orthopedics OPD with complaints of old malunited femur fracture and associated bony deformity, and unable to walk and multiple bony fracture without no history of trauma. On examination there were deformity present in lower limb and old malunited femur fracture left side present. His sclera was normal in color, with no hearing deficit and no rhizomelia. The child's bones were weak, and the cortex was thin, so the child was treated with intravenous zoledronate. The child was planned for surgical intervention based on the work of Sofield and Millar, involving multiple osteotomies, realignment of fragments, and medullary nail fixation for long bones such as the femur, tibia. The Fassier-Duval telescoping rod is designed for children with osteogenesis imperfecta to prevent and stabilize fractures and correct deformities of long bones during growth. The advantage of this expandable rod is its easy placement and better fixation in the physis of long bones.

**Figure 1:**
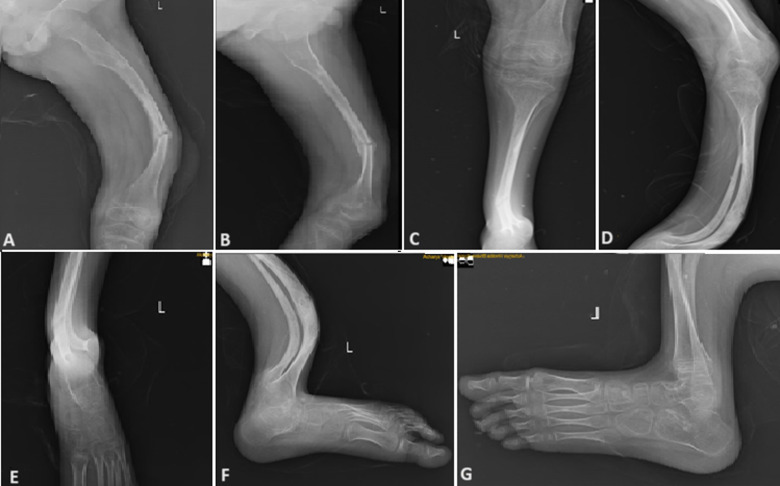
(A, B) X-ray suggestive of anterior bowing deformity of femur with old malunited femur fracture left side; (C, D) X-ray suggestive of anterior bowing deformity of both tibia and fibula left side; (E, F, G) X-ray suggestive of abnormal growths of tarsal bones present

